# Saikosaponin-d affects the differentiation, maturation and function of monocyte-derived dendritic cells

**DOI:** 10.3892/etm.2014.1568

**Published:** 2014-02-20

**Authors:** ZUO-LIN YING, XIAO-JIE LI, HONG DANG, FENG WANG, XIAO-YAN XU

**Affiliations:** 1 Department of Dermatology, Shanghai First People’s Hospital, School of Medicine, Shanghai Jiao Tong University, Shanghai 200080, P.R. China; 2 Experimental Research Center, Shanghai First People’s Hospital, School of Medicine, Shanghai Jiao Tong University, Shanghai 200080, P.R. China

**Keywords:** saikosaponin-d, human papillomavirus, dendritic cells, differentiation, maturation

## Abstract

Saikosaponin-d (Ssd) is a triterpenoid saponin derived from *Bupleurum falcatum* L., which has been shown to exhibit a variety of pharmacological properties, including anti-inflammatory, antibacterial and antiviral properties. The aim of the present study was to investigate the effect of Ssd on the differentiation, maturation and function of human monocyte-derived dendritic cells (DCs) isolated from condylomata acuminata patients. The results of the present study demonstrated that Ssd reduced the differentiation of DCs, as evidenced by decreased expression levels of cluster of differentiation (CD)1a, CD80 and CD86 molecules and increased CD14 expression. Expression levels of the mannose receptor and CD32 were also significantly elevated, which was associated with enhanced fluorescein isothiocyanate-dextran endocytic activity. Furthermore, Ssd treatment promoted DC maturation by increasing the expression levels of CD40, CD83, CD80 and CD86. In addition, the function of mature DCs, including the secretion of IL-12 and the stimulation of lymphocyte proliferation, was significantly increased following Ssd administration. In conclusion, the present study indicated that Ssd exhibited immunomodulatory effects and may be a novel potent chemopreventive drug candidate for the treatment of condylomata acuminata.

## Introduction

Human papillomavirus (HPV) causes various venereal infections, including condylomata acuminata, which are frequently asymptomatic but occasionally cause clinical symptoms of anogenital pruritus and burning sensations (1,2). The current methods for treating condyloma acuminata include cryotherapy, immune stimulation with imiquimod and laser therapy (3). However, HPV-mediated lesions are complex to eradicate, thus, it is imperative to identify novel and effective drugs for the treatment of condylomata acuminata.

Saikosaponin-d (Ssd) is one of the major triterpenoid saponins derived from *Bupleurum falcatum* L., which is commonly prescribed for inflammatory and infectious diseases in China, Japan and other Asian countries (4,5). Previous studies have identified that Ssd exhibits immunomodulatory, anti-inflammatory and antiviral activities, thus, may be a promising chemotherapeutic drug candidate for condylomata acuminata (6,7). Dendritic cells (DCs) have been identified as the most potent antigen-presenting cells that are effective initiators of the immune response to diseases resulting from viral infection (8). Results from previous investigations have indicated that DCs are critical in the induction and regulation of immune responses (9,10). Previous studies have identified that human peripheral blood mononuclear cells (PBMCs) can be induced into DCs in the presence of granulocyte-macrophage colony-stimulating factor (GM-CSF) and interleukin (IL)-4, enabling the collection of a large quantity of DCs for use in clinical application (11,12). Following culturing for five to seven days, PBMCs can be induced to differentiate into immature DCs, which may subsequently be induced to differentiate into mature DCs using inflammatory factor stimulators, including lipopolysaccharide (LPS) (13). As mature DCs alone are capable of activating the immune system and protecting the body against infected pathogens, it is critical to identify effective factors that promote the maturation of DCs.

In the present study, monocyte-derived DCs obtained from condylomata acuminata patients were used to evaluate the effect of Ssd on the modulation of DC differentiation, maturation and function. The aim of the present study was to identify novel and effective drugs that are involved in immunomodulation and that may be used to treat condylomata acuminata.

## Materials and methods

### Chemicals

Ssd (>95% purity, identified by high-performance liquid chromatography), GM-CSF, IL-4 and LPS were purchased from Sigma-Aldrich (St. Louis, MO, USA). Ssd was dissolved in dimethyl sulfoxide and stored at room temperature. RPMI 1640, fetal bovine serum (FBS) and penicillin-streptomycin were obtained from Gibco-BRL (Carlsbad, CA, USA). Fluorescein isothiocyanate (FITC)-dextran, anti-human cluster of differentiation (CD)83, CD80, CD86, CD14, CD1a, CD32, CD40 and mannose receptor (MR) were purchased from BD Pharmingen (San Diego, CA, USA).

### Isolation and generation of PBMCs

PBMCs were isolated using Ficoll density gradient centrifugation, according to the manufacturer’s instructions. Mononuclear cells were incubated for 3 h in six-well plates in RPMI 1640 buffer that was supplemented with 10% FBS at 37°C in a humidified atmosphere with 5% CO_2_. The nonadherent cells were removed by gentle washing and the remaining adherent monocytes were cultured in RPMI 1640 with GM-CSF and IL-4 for five days to generate immature DCs. Maturation was generated by stimulation of the immature DCs with 10 ng/ml LPS for a further 48 h. Ssd at varying concentrations (5, 10 and 20 μM) was added to the culture for five days to investigate the effect on DC differentiation. Ssd was subsequently added to the five-day cultured immature DCs to evaluate the effect on the maturation of the DCs. The study was approved by the ethics committee of Shanghai Jiao Tong University, Shanghai, China. Written informed patient consent was obtained from the patient.

### Flow cytometry

Expression levels of cell surface markers were detected via flow cytometry. Cells were harvested following a five or seven-day culture and were resuspended in phosphate-buffered saline. The expression levels of CD83, CD80, CD86, CD14, CD1a, CD32, CD40 and MR markers were measured using flow cytometry; the expression rate of the markers was determined by CellQuest software (BD Biosciences, San Jose, CA, USA).

### Detection of DC endocytic activity

Endocytic activity of the DCs was measured via FITC-dextran uptake and determined using flow cytometry. Cells at a density of 4×10^6^ cells/sample were incubated in medium containing FITC-dextran at 37°C for 2 h. The uptake of FITC-dextran by the cells was calculated using flow cytometry and the mean fluorescence density represented the uptake ability of the DCs.

### Mixed lymphocyte reactions (MLRs)

Responder cells were purified with allogeneic CD4^+^ T cells using a magnetic-activated cell sorting CD4^+^ T cell isolation kit (Miltenyi Biotec, Auburn, CA, USA). The allostimulatory capacity of irradiated DCs (30 Gy) was measured at different stimulator/responder cell ratios in 96-well flat-bottom plates. Thymidine incorporation was measured via standard liquid scintillation counting.

### Determination of cytokine secretion by DCs

Supernatants were collected and stored at −80°C, until required for cytokine analysis. ELISA (R&D Systems, Minneapolis, MN, USA) was used to determine the expression levels of IL-12 and was performed according to the manufacturer’s instructions. Absorbance was measured at 450 nm using a microplate reader and IL-12 content was determined according to the standard curve.

### Statistical analysis

Data are expressed as the mean ± SEM and statistical analysis was conducted using SPSS 10.0 software (SPSS, Inc., Chicago, IL, USA). Comparisons between groups were performed with analysis of variance and P<0.05 was considered to indicate a statistically significant difference.

## Results

### Effects of Ssd on the differentiation of monocyte-derived DCs

**Figure 1:**
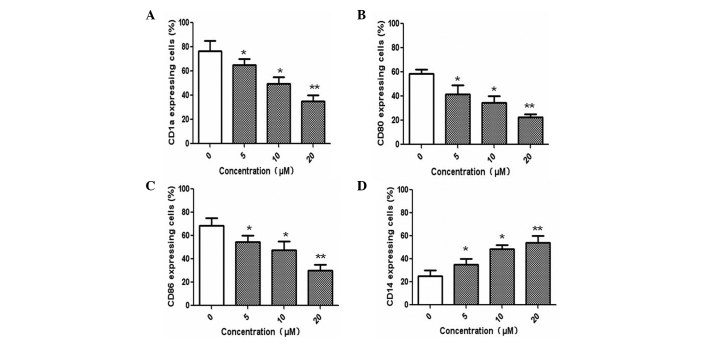
Effects of Ssd on the differentiation of monocyte-derived DCs. Human monocytes were incubated with GM-CSF, IL-4 and various concentrations of Ssd (5, 10 and 20 μM) for five days. Flow cytometry was used to analyze the expression levels of (A) CD1a, (B) CD80, (C) CD86 and (D) CD14. ^*^P<0.05 and ^**^P<0.01 vs. 0 μM Ssd. GM-CSF, granulocyte-macrophage colony-stimulating factor; IL, interleukin; Ssd, saikosaponin-d; CD, cluster of differentiation; DCs, dendritic cells.

To investigate the effect of Ssd on DC differentiation, monocytes were cultured in the presence of GM-CSF and IL-4 with various concentrations of Ssd (5, 10 and 20 μM). The expression levels of CD80, CD86, CD14 and CD1a were determined by flow cytometry. As shown in Fig. 1, treatment of monocytes with Ssd resulted in decreased expression levels of CD1a, CD80 and CD86 in a concentration-dependent manner. However, the expression of CD14 was significantly upregulated following Ssd stimulation, indicating that Ssd inhibited the differentiation of DCs from monocytes in a concentration-dependent manner.

### Effects of Ssd on the endocytic activity of immature DCs

**Figure 2:**
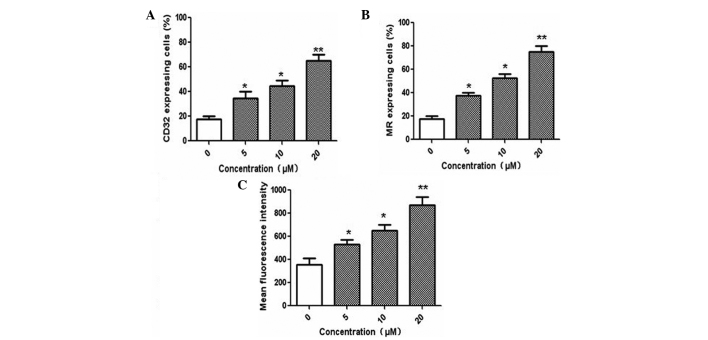
Effects of Ssd on the endocytic activity of immature DCs. DCs were differentiated from monocytes with GM-CSF and IL-4 with various concentrations of Ssd (5, 10 and 20 μM). Flow cytometry was conducted to determine the expression levels of (A) CD32 and (B) MR, as well as (C) mean fluorescence intensity. ^*^P<0.05 and ^**^P<0.01 vs. 0 μM Ssd. DCs, dendritic cells; GM-CSF, granulocyte-macrophage colony-stimulating factor; IL, interleukin; Ssd, saikosaponin-d; CD, cluster of differentiation; MR, mannose receptor.

Immature DCs are capable of effectively capturing and processing antigens. Therefore, the effects of Ssd on the endocytic activity of immature DCs were examined. The results indicated that Ssd treatment significantly enhanced the expression levels of molecules involved in antigen uptake, including CD32 and MR, which was consistent with the inhibition of Ssd on the differentiation of monocyte-derived DCs (Fig. 2A and B). In addition, flow cytometric analysis demonstrated that Ssd increased the uptake of FITC-dextran, indicating the enhanced endocytic activity of the immature DCs (Fig. 2C).

### Effects of Ssd on the maturation of monocyte-derived DCs

**Figure 3:**
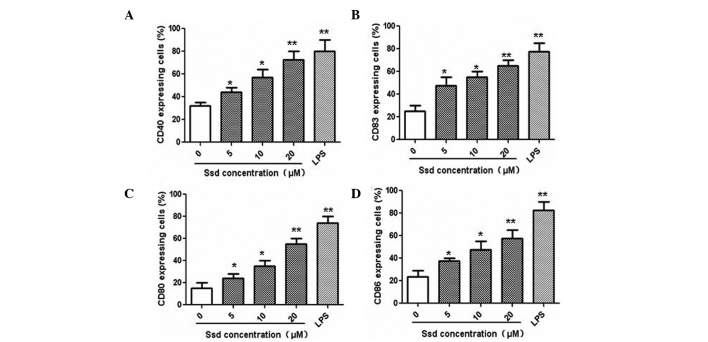
Effects of Ssd on the maturation of monocyte-derived DCs. Immature DCs were incubated with various concentrations of Ssd (5, 10 and 20 μM) or 10 ng/ml LPS for 48 h and the expression levels of (A) CD40, (B) CD83, (C) CD80 and (D) CD86 were determined. ^*^P<0.05 and ^**^P<0.01 vs. 0 μM Ssd. DC, dendritic cell; IL, interleukin; Ssd, saikosaponin-d; LPS, lipopolysaccharide; CD, cluster of differentiation.

Following five days of culturing with GM-CSF and IL-4, immature DCs were able to further differentiate into mature DCs via stimulation with LPS. On day five, the immature DCs were incubated with various concentrations of Ssd (5, 10 and 20 μM) or 10 ng/ml LPS for an additional 48 h. The results demonstrated that Ssd promoted the maturation of the immature DCs in a dose-dependent manner by increasing CD40 and CD83 expression, as well as by upregulating CD80 and CD86 expression (Fig. 3). These results indicated that Ssd promoted the maturation of immature DCs in a concentration-dependent manner.

### Effects of Ssd on the terminal function of mature DCs

**Figure 4:**
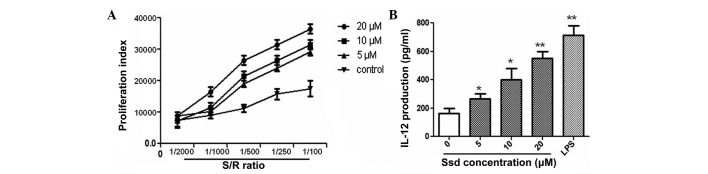
Effects of Ssd on the terminal function of mature DCs. Monocytes were cultured with GM-CSF and IL-4 for five days and incubated with Ssd or LPS for a further 48 h. (A) Thymidine incorporation assay was used to measure lymphocyte proliferation. (B) IL-12 expression levels were measured with ELISA. ^*^P<0.05 and ^**^P<0.01 vs. 0 μM Ssd. S/R, stimulator/responder cell; GM-CSF, granulocyte-macrophage colony-stimulating factor; IL, interleukin; Ssd, saikosaponin-d; DCs, dendritic cells; LPS, lipopolysaccharide.

Mature DCs have been shown to play a critical role in antigen presentation and the stimulation of lymphocyte proliferation with MLRs. The present study demonstrated that lymphocyte proliferation significantly increased in a dose-dependent manner following Ssd stimulation (Fig. 4A). In addition, the effect of Ssd on the secretion of IL-12 by mature DCs was measured using ELISA. Compared with the control group, IL-12 expression in the mature DCs treated with Ssd significantly increased in a dose-dependent manner (Fig. 4B).

## Discussion

HPV infection is prevalent around the world and can lead to benign lesions, such as condyloma acuminata, and malignant lesions, including cervical cancer (14). DCs are critical for antigen presentation and defense of the antiviral host, thus, the infection of immature DCs by numerous types of virus impairs maturation and reduces the DC ability to stimulate lymphocyte proliferation (15). A traditional drug, Ssd, has been reported to possess immunoodulatory, anti-inflammatory and antiviral properties; therefore, Ssd is commonly prescribed to treat inflammatory and infectious diseases (4). In the present study, the effects of Ssd on the differentiation, maturation and terminal function of monocyte-derived DCs isolated from condyloma acuminata patients, were investigated.

DCs have two stages, immature and mature. Immature DCs possess a weak ability to stimulate lymphocyte proliferation, but can effectively capture and process antigens (16). The present study demonstrated that Ssd treatment reduced the differentiation of DCs, as shown by decreased expression levels of CD1a, which is a characteristic DC-associated molecule (17). The expression of molecules involved in antigen presentation, including CD80 and CD86, decreased while CD14 expression (a typical marker of monocyte/macrophage not normally present on DCs) was markedly increased (18). These observations indicate that Ssd inhibits the differentiation of DCs from monocytes in a concentration-dependent manner. In addition, the immature DC is characterized by a high capacity for antigen uptake and processing, thus, is associated with high expression levels of molecules involved in antigen uptake, including CD32 and MR (19). Flow cytometric analysis showed that treatment with Ssd increased the uptake of FITC-dextran, as well as the expression of CD32 and MR, indicating the enhanced endocytic activity of immature DCs. These results are consistent with the hypothesis that Ssd inhibits the differentiation of DCs.

The unique ability of DCs to activate lymphocyte proliferation is dependent on the stage of maturation, cytokine secretion and expression of costimulatory molecules, including CD86 and CD80 (20,21). Viral and bacterial products, as well as inflammatory cytokines, are able to initiate DC maturation to increase the expression of costimulatory molecules and the capacity of DCs to promote lymphocyte proliferation (22–24). Mature DCs reduce their endocytic capacity, but enhance chemokine and inflammatory cytokine production and become mobile to enable the delivery of pathogen-derived antigens for lymphocyte activation (25). Considering the importance of mature DCs in primary immune responses, the induction of DC maturation is critical to defend against viral infections. In the present study, immature DCs were treated with Ssd or LPS for 48 h to study the effect of Ssd on DC maturation. The expression of CD83, a typical marker of DC maturation (26), was shown to significantly increase in a dose-dependent manner. The expression levels of costimulatory molecules, including CD80, CD86 and CD40, were also elevated following Ssd treatment. Furthermore, consistent with the changes of phenotype, Ssd stimulation significantly promoted lymphocyte proliferation and IL-12 expression in mature DCs. Therefore, these results indicate that Ssd promotes DC maturation in a concentration-dependent manner.

In conclusion, the results of the present study indicate that Ssd exhibits an immunomodulatory effect and therefore may be a novel potent chemopreventive drug candidate for the treatment of condylomata acuminata.
